# Phenotypic antibiotic resistance of *Mycoplasma genitalium* and its variation between different macrolide resistance-associated mutations

**DOI:** 10.1093/jac/dkae430

**Published:** 2024-12-04

**Authors:** T A Doelman, N Adriaens, B M Westerhuis, S M Bruisten, C E Vergunst, F M Bouwman, A P van Dam

**Affiliations:** Department of Medical Microbiology, Amsterdam UMC Location University of Amsterdam, Amsterdam, The Netherlands; Infectious Diseases, Amsterdam Institute for Immunology and Infectious Diseases, Amsterdam, The Netherlands; Infectious Diseases, Amsterdam Institute for Immunology and Infectious Diseases, Amsterdam, The Netherlands; Department of Infectious Diseases, Public Health Service of Amsterdam, Amsterdam, The Netherlands; Department of Infectious Diseases, Public Health Service of Amsterdam, Amsterdam, The Netherlands; Infectious Diseases, Amsterdam Institute for Immunology and Infectious Diseases, Amsterdam, The Netherlands; Department of Infectious Diseases, Public Health Service of Amsterdam, Amsterdam, The Netherlands; Department of Infectious Diseases, Public Health Service of Amsterdam, Amsterdam, The Netherlands; Department of Dermatology, Noordwest Ziekenhuisgroep location Den Helder, Den Helder, The Netherlands; Department of Infectious Diseases, Public Health Service of Amsterdam, Amsterdam, The Netherlands; Department of Medical Microbiology, Amsterdam UMC Location University of Amsterdam, Amsterdam, The Netherlands; Infectious Diseases, Amsterdam Institute for Immunology and Infectious Diseases, Amsterdam, The Netherlands; Department of Infectious Diseases, Public Health Service of Amsterdam, Amsterdam, The Netherlands

## Abstract

**Objectives:**

*Mycoplasma genitalium*, a sexually transmitted bacterium, faces increasing antibiotic resistance, particularly to azithromycin. However, presence of macrolide resistance-associated mutations (MRAMs) does not evidently implicate azithromycin treatment failure. This study aimed to establish an *in vitro* co-culture system of *M. genitalium* isolates and perform phenotypic susceptibility testing for different antibiotics, focusing on azithromycin to evaluate genotypic and phenotypic resistance across MRAMs.

**Methods:**

Urine specimens testing positive for *M. genitalium* via nucleic acid amplification were co-cultured with Vero cells. Phenotypic susceptibility testing was performed for eight antibiotics. Growth inhibition and MIC of *M. genitalium* by azithromycin were compared across different MRAMs.

**Results:**

*M. genitalium* was cultured from 20/40 (50.0%) positive urine samples, with phenotypic susceptibility tested in a subset. MICs ranged as follows: azithromycin (0.008–>32 mg/L), levofloxacin (1–4 mg/L), moxifloxacin (<0.25–1 mg/L), sitafloxacin (<0.032–0.25 mg/L), minocycline (<0.25–1 mg/L), doxycycline (<0.125–2 mg/L), spectinomycin (<2.5–>25 mg/L) and lefamulin (<0.004–0.063 mg/L). Isolates with A2058T demonstrated 24-, 7-, 15- and 12-fold increases in growth inhibition compared with A2058G at azithromycin concentrations of 4, 8, 16 and 32 mg/L, respectively (*P < *0.01). MRAMs ranked from low to high impact on MIC range were as follows: wildtype (0.008–0.016), A2058T (8–32), A2059G (≥32) and A2058G (>32).

**Conclusions:**

This study revealed that *M. genitalium* isolates vary in azithromycin-induced growth inhibition across MRAMs, potentially explaining differences in clinical treatment efficacy. Phenotypic susceptibility testing for other antibiotics demonstrated relatively low MICs. Future studies should incorporate clinical treatment efficacy and symptom severity to optimize treatment for *M. genitalium*.

## Introduction

*Mycoplasma genitalium* is a sexually transmitted bacterium recognized to cause non-gonococcal urethritis (NGU) in men^1–3^ and cervicitis in women.^4,5^ In the Netherlands, the prevalence of *M. genitalium* among patients visiting the general practitioner (GP) is 6.7% in females and 3.7% in males.^6^ The prevalence of infection in sexually transmitted infection (STI) clinics averages 13.6% for all patients, with the highest prevalence of 20.1% observed in men who have sex with men.^7^ A significant concern regarding *M. genitalium* infections is the increasing antibiotic resistance due to its rapid molecular mutation rate.^8^

Due to the absence of a cell wall and the rapid mutation rate, treatment options for *M. genitalium* infections have become increasingly limited. Resistance to azithromycin, the first-line treatment, has been observed in 66% and 73% of *M. genitalium* isolates among visitors of STI clinics and GPs, respectively, in the Netherlands.^6,7^ Regarding second-line treatment with moxifloxacin, a fluoroquinolone resistance prevalence of 7% has been reported among STI clinic visitors.^9^ If both treatment options fail, the 2021 European guidelines recommend pristinamycin as a third-line therapy, which is currently not readily available in the Netherlands.^10^ Minocycline is an alternative option, with a reported cure rate of 71%.^11^ Research into new potential antibiotics via phenotypic susceptibility testing has been limited, primarily due to the challenges associated with *in vitro* growth of *M. genitalium.*^12^

With the rise of resistant isolates and the limitations of phenotypic susceptibility testing, genotypic resistance testing for macrolides and fluoroquinolones has become the standard method for assessment of resistance. Fluoroquinolone treatment failure due to resistance is likely to occur when quinolone resistant-associated mutations in the *parC* gene, and/or the *gyrA* gene are identified.^13,14^ Macrolide resistance-associated mutations (MRAMs) in *M. genitalium* are characterized as single nucleotide polymorphisms (SNPs) at nucleotide positions 2058 and 2059 of the 23S rRNA gene (*E. coli* numbering). The most commonly observed SNPs in *M. genitalium* are A2058G and A2059G, which have been previously associated with phenotypic and clinical treatment failure of azithromycin.^15–17^ For other SNPs, including A2058T, the link between genotypic and phenotypic antibiotic resistance has not yet been evaluated, mainly due to the scarcity of *M. genitalium* isolates containing this mutation. However, the prevalence of A2058T has increased in the Netherlands during recent years,^18,19^ with ∼12% of the *M. genitalium* infections now harbouring this mutation.^20^ This rise in prevalence highlights the need for further investigation into the phenotypic antibiotic resistance associated with this MRAM SNP. Recent studies by Braam *et al.*^20^ and Bachman *et al.*^21^ aimed to determine prevalence of *M. genitalium* among men with NGU and assess symptom resolution 2 weeks after azithromycin treatment. Findings indicated that symptom resolution could occur in 54%^20^ and 68%^21^ of the NGU patients infected with *M. genitalium* following azithromycin therapy, despite the detection of an MRAM SNP. This suggests that genotypic macrolide resistance does not inevitably lead to clinical azithromycin treatment failure. In this study, we established an *in vitro* co-culture system using *M. genitalium* isolates from STI clinic visitors in the Netherlands and performed phenotypic susceptibility testing for a number of relevant and available antibiotics to re-evaluate and explore alternative therapeutic options. We specifically focused on azithromycin to assess the relationship between genotypic and phenotypic macrolide resistance across different MRAM SNPs.

## Materials and methods

### Ethics statement

This study did not involve data collection from human subjects and was therefore exempted from ethical clearance.

### Collection of *M. genitalium* isolates

At the Public Health Service of Amsterdam, the Netherlands, first-void urine samples were randomly collected between November 2021 and March 2023. Upon collection, samples were anonymized and tested for *M. genitalium* using a transcription mediated amplification (TMA) assay (Aptima *Mycoplasma genitalium* assay; Hologic Inc. San Diego, CA, USA).^22^ Positive samples were centrifuged within 24 h post collection at either 10 000 × **g** for 15 min or 21 000 × **g** for 20 min. The pellet was resuspended in 1 mL Eagle’s Minimal Essential Medium (EMEM, Gibco, Montana, USA) and frozen at −80°C. TMA-positive urine samples were re-evaluated for *M. genitalium* by qPCR and genotyped for MRAM SNPs by single-target qPCR assays.^22–24^

### Cell culture of *M. genitalium* isolates

Vero cells (ATCC CCL-81) were cultured in EMEM supplemented with 8% FBS (Gibco or Sigma). The *M. genitalium* reference strain G37 was used for validation of the culture system. A modified version of the protocols by Hamasuna *et al.*^25^ and Wood *et al*.^26^ was utilized to culture the *M. genitalium* isolates. Vero cells (1 × 10^5^) were seeded in 25-cm^2^ tissue culture flasks containing 5 mL of EMEM supplemented with 8% FBS, 100 IU/mL of penicillin G, 50 μg/mL polymyxin B and 30 μg/mL collistin (antibiotics obtained from Sigma, St. Louis, MO, USA). FBS from Gibco (cat. No. 10270-106) or Sigma (cat. No. F9665) containing a minimum alpha globulin concentration of 16 g/L was required to facilitate *M. genitalium* growth. Processed urine samples were thawed in warm water, and 300 μL was used as inoculum. Following inoculation, 100 μL of both the processed urine specimen and the suspension was obtained for qPCR to assess *M. genitalium* load in the inoculum and at day zero, respectively. Cultures were incubated for two or 3 weeks at 37°C in 5% CO_2_. After incubation, Vero cells were scraped and 100 μL of the suspension was harvested for *M. genitalium* qPCR. Growth was quantified by comparing the *M. genitalium* load at harvest with the load measured at day zero. Based on the quantified growth, up to 2 mL suspension was used for sub-culturing. Following the initial passage, samples were sub-cultured without antibiotics to minimize growth inhibition, with aliquots stored at −80°C after each passage. Cultures were passaged until reaching a load of >1 × 10^8^ genome equivalent (geq)/mL. Subsequently, cultures were either used for immediate testing or stored at −80°C.

### DNA isolation and qPCR assay of cultured isolates

After inoculation, DNA isolation was performed using 20% chelex 100 slurry (Bio-Rad Laboratories, Hercules, CA, USA).^27^ The MgPa Taqman real-time PCR^28^ for quantifying *M. genitalium* load in cultured samples was modified to include an internal amplification control (IAC) targeting synthetic DNA. A standard curve was generated using purified whole genome *M. genitalium* DNA (Vircell, Granada, Spain), and the curve formula was used to calculate *M. genitalium* load. Briefly, 4 μL of isolated DNA and 1 μL containing 5–50 copies of synthetic internal control DNA^24^ was added to 15 μL platinum Q supermix (Thermo Fisher Scientific, Waltham, MA, USA), 625 nM of MgPa primers,^29^ 400 nM of IAC primers^24^ and 225 nM of both MgPa and IAC probes, bringing the total volume to 31 μL. Each reaction mix was aliquoted into three wells, using 9 μL per well, to allow for triplicate testing using the previously described PCR programme.^22^

### Phenotypic antibiotic susceptibility testing

Azithromycin (Sigma, St. Louis, MO, USA), levofloxacin (Santa Cruz Biotechnology, Dallas, TX, USA), moxifloxacin (Sigma), sitafloxacin (Targetmol Chemicals Inc., Boston, MA, USA), lefamulin (MedChemExpress, Monmouth Junction, NJ, USA), doxycycline (Sigma), minocycline (Santa Cruz Biotechnology) and spectinomycin (Thermo Fisher Scientific, Waltham, MA, USA) were used for phenotypic antibiotic susceptibility testing.

MICs of *M. genitalium* isolates were determined using an adapted protocol from Wood *et al.*^17^ Briefly, 8 × 10^3^ Vero cells were seeded in 12-well plates containing 1 mL of EMEM supplemented with 8% FBS. *M. genitalium*, sourced from frozen aliquots or 25-cm^2^ tissue culture flasks, was added in quantities of 0.6–3 × 10^6^ per 0.5 mL of medium per well. A 50 μL sample was collected from one of the wells and mixed with 50 μL of medium for DNA extraction using 20% chelex 100 slurry in order to assess *M. genitalium* load at day zero. At the onset of log-phase growth, following a seven-day incubation period, 1.5 mL of medium containing specified antibiotic dilutions was introduced to the culture. Previous experiments demonstrated an absence of *M. genitalium* growth between Days 0 and 7 in 12-well plates (Figure S1, available as Supplementary data at *JAC* Online). Based on the lack of observable growth during this initial week, it was decided to add antibiotics on Day 7 rather than at an earlier time point. Three concentrations of each antibiotic were tested (Table 1), based on prior studies.^14,15,26–29^ All isolates containing MRAMs were evaluated using a 2-fold serial dilution of azithromycin. Each concentration and the control without antibiotics were tested in triplicate. After 28 days, wells were scraped and 100 μL was harvested for DNA extraction and qPCR. The inhibition percentage was determined by comparing the load of *M. genitalium* with and without antibiotics.^25^ MIC was defined as the lowest antibiotic concentration achieving >99% inhibition of *M. genitalium* growth in a specific isolate. MIC_50_ and MIC_90_ were described as the antibiotic concentrations required to inhibit 99% of *M. genitalium* growth in 50% and 90% of the isolates, respectively.

**Table 1.** Antibiotic concentrations assessed during phenotypic susceptibility testing

Antibiotic class	Antibiotic	Low concentration (mg/L)	Intermediate concentration (mg/L)	High concentration (mg/L)
Macrolides	Azithromycin	0.016	−/4/8^a^	8/16^a^
Fluoroquinolones	Levofloxacin	0.5	1	4
	Moxifloxacin	0.25	1	4
	Sitafloxacin	0.032	0.25	1
Tetracyclines	Minocycline	0.25	0.5	1
	Doxycycline	0.125	0.5	2
Aminocyclitol	Spectinomycin	2.5	12.5	25
Pleuromutilin	Lefamulin	0.004	0.016	0.063

^a^Intermediate and high concentrations of azithromycin varied based on MRAM SNP and the testing round.

The phenotypic antibiotic susceptibility assay for azithromycin was repeated with additional constraints in order to compare the growth inhibition of *M. genitalium* between MRAM SNPs. A minimum of three isolates per MRAM SNP and wildtype were cultured and frozen in five aliquots for at least 24 h. The MIC assay was repeated using these aliquots to ensure consistency in initial conditions.

### Statistical analysis

Significant differences in growth inhibition among *M. genitalium* isolates with different MRAM SNPs were assessed using the Kruskal–Wallis test (*P* < 0.05). SPSS Statistics (version 26.0, IBM, Armonk, NY, USA) was used for statistical analysis, and GraphPad Prism (version 8, GraphPad Software, La Jolla California, USA) was used to create figures.

## Results

### *M. genitalium* isolate collection, MRAM distribution and culture

A total of 601 urine specimens were collected, of which 71 (11.8%) and 46 (7.7%) tested positive for *M. genitalium* by TMA and MgPa qPCR, respectively. Genotyping for MRAM SNP revealed that 6/46 (13.0%) of the qPCR-positive specimens were genotypically wildtype, 11/46 (23.9%) harboured the A2058G SNP, 14/46 (30.4%) the A2059G SNP, 8/46 (17.4%) the A2058T SNP, and 7/46 (15.2%) were untypable. Overall, 40/46 *M. genitalium* MgPa qPCR-positive samples were cultured, of which 20/40 (50.0%) demonstrated successful growth. Among the successful cultures, 3/20 (15.0%) were genotypically wildtype, 3/20 (15.0%) harboured the A2058G SNP, 8/20 (40.0%) the A2059G SNP and 6/20 (30.0%) the A2058T SNP. The initial *M. genitalium* loads for 18 of the 20 cultured isolates ranged from 2645 to 1.0 × 10^6^ geq/mL, while 2 isolates exhibited loads below the quantifiable threshold of 2500 geq/mL.

### MIC determination by phenotypic antibiotic susceptibility testing

Table 2 presents the range, MIC_50_ and MIC_90_ for the eight antibiotics evaluated by phenotypic antibiotic susceptibility testing against *M. genitalium* isolates. Among the fluoroquinolones, sitafloxacin demonstrated the highest activity, with an MIC_50/90_ of 0.032/0.25 mg/L. Moxifloxacin followed, with an MIC_50/90_ of 0.5/1 mg/L. All cultured isolates demonstrated high susceptibility to lefamulin, with only one strain showing an elevated MIC of 0.063 mg/L. Detailed MIC values per strain are provided in Table S1.

**Table 2.** Range, MIC_50_ and MIC_90_ values of various antibiotics against *M. genitalium* isolates

Antibiotic class	Antibiotic	No. of isolates	Range (mg/L)	MIC_50_ (mg/L)	MIC_90_ (mg/L)
Macrolides	Azithromycin	16	0.008–>32	32	>32
Fluoroquinolones	Levofloxacin	12	1–4	4	>4
	Moxifloxacin	11	<0.25–1	1	1
	Sitafloxacin	12	<0.032–0.25	0.032	0.25
Tetracyclines	Minocycline	11	<0.25–1	0.5	>1
	Doxycycline	11	<0.125–2	0.5	2
Aminocyclitol	Spectinomycin	12	<2.5–>25	12.5	>25
Pleuromutilin	Lefamulin	11	<0.004–0.063	0.004	0.016

**Figure 1.**
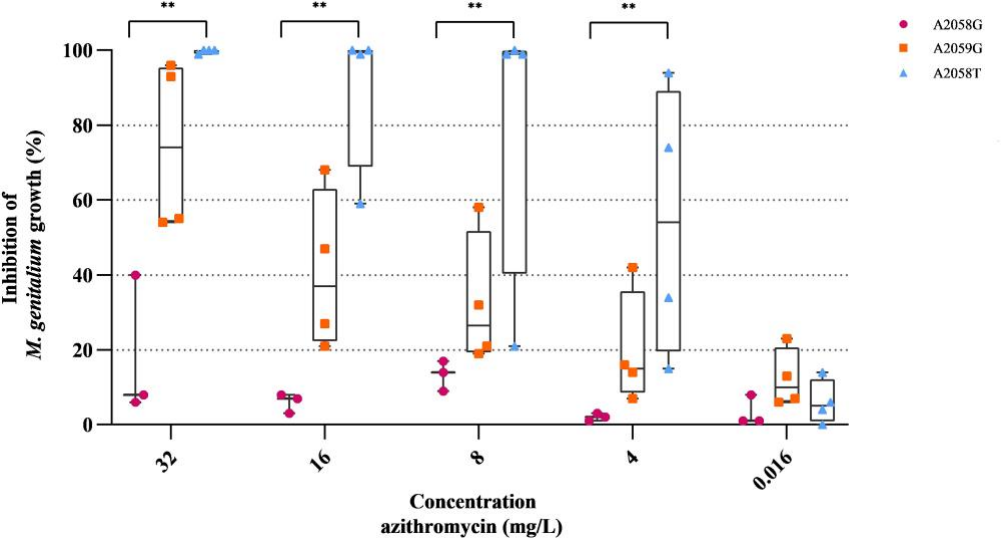
Inhibition of *M. genitalium* growth at various azithromycin concentrations. Included *M. genitalium* isolates harboured either the A2058G (*n* = 3), A2059G (*n* = 4) or A2058T (*n* = 4) MRAM SNP. The median percentages of growth inhibition are presented, including interquartile range (box) and minimum/maximum (whiskers). The Kruskal–Wallis test was performed to indicate significant differences in inhibition of *M. genitalium* growth between isolates (**, *P* < 0.01). All genotypic wildtype isolates (*n* = 4) demonstrated 100% growth inhibition at all tested azithromycin concentrations (data not shown).

Azithromycin MIC_50_ and MIC_90_ values for 16 isolates were determined to be 8 and >32 mg/L, respectively. More extensive susceptibility testing for azithromycin was successfully performed on 15/16 isolates. Genotypic wildtype isolates (*n* = 4) demonstrated an MIC range of 0.008–0.016 mg/L, whereas isolates containing MRAM SNPs showed MICs ranging from 8 to >32 mg/L. *M. genitalium* growth inhibition of isolates harbouring A2058G (*n* = 3), A2059G (*n* = 4) or A2058T (*n* = 4) SNPs at various azithromycin concentrations was compared (Figure 1). For A2058G isolates, growth inhibition of *M. genitalium* was below 20% across all tested concentrations, with the exception of one isolate at 32 mg/L. In contrast, isolates harbouring the A2058T SNP demonstrated significant 24-, 7-, 15- and 12-fold increases in median growth inhibition compared with the A2058G SNP at azithromycin concentrations of 4, 8, 16 and 32 mg/L, respectively (*P < *0.01). Specifically, three out of four A2058T isolates showed >99% growth inhibition at 8 mg/L azithromycin. One A2058T isolate displayed reduced sensitivity, with inhibition patterns comparable to those of A2059G isolates. All A2058T isolates were fully inhibited at 32 mg/L. Growth inhibition for A2059G isolates was greater compared with those with the A2058G SNP but less than that observed in A2058T isolates; however, these differences were not statistically significant. All genotypic wildtype isolates (*n* = 4) demonstrated 100% growth inhibition at all tested azithromycin concentrations (data not shown). The range, MIC_50_ and MIC_90_ values of azithromycin against *M. genitalium* isolates are summarized in Table 3, categorized according to MRAM SNP. The SNPs ranked from low to high impact on MIC range are as follows: wildtype (0.008–0.016), A2058T (8–32), A2059G (≥32) and A2058G (>32). SNPs remained consistent before and after susceptibility testing, as confirmed by MRAM qPCR. No new mutations were induced at nucleotide positions 2058 and 2059 of the 23S rRNA gene throughout the course of these experiments.

**Table 3.** Range, MIC_50_ and MIC_90_ values of azithromycin against *M. genitalium* isolates categorized per MRAM SNP

MRAM SNP	No. of isolates	Range (mg/L)	MIC_50_ (mg/L)	MIC_90_ (mg/L)
Wildtype	4	0.008–0.016	≤0.016	≤0.016
A2058G	3	>32	>32	>32
A2059G	5	≥32	>32	>32
A2058T	4	8–32	8	32

## Discussion

In this study, we established an *in vitro* co-culture system of *M. genitalium* isolates from STI clinic visitors in the Netherlands and performed phenotypic antibiotic susceptibility testing for a number of relevant and available antibiotics to re-evaluate and explore alternative therapeutic options. We specifically focused on azithromycin to assess the relationship between genotypic and phenotypic macrolide resistance across different MRAM SNPs.

We successfully cultured 20 *M. genitalium* isolates from urine specimens and performed phenotypic azithromycin susceptibility testing on a subset. Results demonstrated a broad spectrum of MIC values for azithromycin, ranging from 0.008 to >32 mg/L, consistent with MIC values reported in other studies.^17,30,31^ Isolates exhibiting high MICs (≥8 mg/L) consistently harboured an MRAM SNP, whereas isolates with low MICs (0.008–0.016 mg/L) were genotypically wildtype. Detailed analysis of individual MRAM SNPs revealed that isolates with MRAM A2058T demonstrated significant increases in median growth inhibition compared with the A2058G SNP at azithromycin concentrations of 4–32 mg/L. MRAMs ordered from low to high impact on MIC range were as follows: wildtype (0.008–0.016), A2058T (8–32), A2059G (≥32) and A2058G (>32).

According to Dutch guidelines, patients diagnosed with NGU receive empirical treatment with a single dose of oral azithromycin.^32^ Interestingly, symptom resolution after treatment with azithromycin has been previously observed, despite the presence of *M. genitalium* harbouring an MRAM. A recent study regarding men with NGU visiting an STI clinic in the Netherlands demonstrated that azithromycin treatment was still clinically effective in 54% of the *M. genitalium* MRAM cases (*n* = 71).^20^ However, this study was limited by the lack of follow-up testing for *M. genitalium* and MRAM SNPs, which precluded differentiation of clinical outcomes attributable to specific SNPs. Our findings support the hypothesis that the efficacy of azithromycin treatment may vary depending on the specific MRAM SNPs present in *M. genitalium* isolates. However, resistance levels in isolates harbouring MRAM SNPs remained substantially higher compared with genotypic wildtype isolates. A supporting hypothesis for the clinical improvement despite presence of *M. genitalium* infection harbouring MRAMs may be linked with the enhanced uptake and delivery of azithromycin by polymorphonuclear (PMN) leukocytes.^33,34^ Due to the attraction of PMNs to inflammatory sites, the local concentration of azithromycin is elevated. In cases of NGU, which is characterized by an enhanced influx of leukocytes at the infection site, the elevated azithromycin concentration may effectively reduce the growth of *M. genitalium*, potentially contributing to improved clinical outcomes. Following a 3-day regimen of 500 mg azithromycin, intracellular concentrations in leukocytes were 60- to 90-fold higher than in plasma and remained elevated through Day 10. The resulting area under the curve values reached ∼800 µg h/mL on Day 3 and 340 µg h/mL on Day 10, exceeding a 10-fold ratio relative to *M. genitalium* isolates with MICs of 8–16 µg/mL.^35^ Additionally, tissue concentrations of azithromycin are higher than those in plasma,^36–38^ likely due to the intracellular accumulation of the drug.^35^ Nevertheless, other factors may also influence treatment outcomes, including the potential for spontaneous clearance of *M. genitalium* infections and the anti-inflammatory properties of macrolides.^39^

In a study by Jensen *et al*.,^16^ seven *M. genitalium* isolates from men who experienced azithromycin treatment failure were cultured, revealing MIC values exceeding 8 mg/L. These elevated MIC values were associated with the SNPs A2058G, A2059G and A2058C. Several other studies have reported similar MIC values of >8 mg/L for azithromycin in *M. genitalium* isolates with these MRAMs, showing a statistically significant correlation with clinical treatment failure.^17,30,31^ In a more recent study,^40^ an *M. genitalium* isolate harbouring the A2058T SNP was tested for azithromycin susceptibility, revealing a lower MIC value of 16 mg/L compared with MICs of >64 mg/L for isolates with A2058G (*n* = 2) and A2059G (*n* = 1) SNPs. To our knowledge, this represents the only reported *M. genitalium* isolate with an A2058T SNP that was subjected to phenotypic azithromycin susceptibility testing, aside from the isolates evaluated in our study. Similar to our findings, there was no available data on *in vivo* treatment efficacy for this isolate, leaving clinical implications undetermined. For haemolytic streptococci and staphylococci, EUCAST MIC breakpoints for azithromycin are set at 0.25 and 2 mg/L, respectively; isolates with MIC values of 8 and 16 mg/L are classified as resistant. Conversely, *Shigella* infections can be successfully treated with azithromycin despite a natural MIC distribution up to 16 mg/L, with resistance mutations typically associated with MICs ≥32 mg/L.^41^ The correlation between MIC values and clinical resistance for *M. genitalium* remains however unclear.

The lack of previously conducted azithromycin susceptibility testing of A2058T isolates may be attributed to their low prevalence until recent years. Isolates harbouring the A2058G and A2059G SNPs are the first and second most common mutations in Europe, Asia and Australia,^19,42–44^ and thus have been the primary focus of culturing and susceptibility testing efforts. Although A2058C and A2059C SNPs have been reported in various regions worldwide, they are not as abundant compared with A2058G and A2059G SNPs.^22,45–48^ With the recent rise in prevalence of A2058T isolates in countries such as the Netherlands,^18^ France^49^ and Belgium,^48^ our study underscores the importance to further investigate the clinical consequence of antibiotic resistance associated with this SNP.

In addition to the extensive phenotypic susceptibility testing for azithromycin, we determined MIC values for a range of other antibiotics to re-evaluate and explore alternative therapeutic options. Based on *in vitro* MIC data only, fourth-generation fluoroquinolones, tetracyclins and lefamulin may be considered as effective treatments for *M. genitalium* infections, including those isolates harbouring MRAM SNPs. These findings align with observations from other studies, which have reported generally low MICs for these antibiotics following antibiotic susceptibility testing of cultured *M. genitalium* isolates.^14,29^ Lefamulin appears to be a particularly promising therapeutic option for *M. genitalium* infections. Similar to our results, a study by Paukner *et al.*^26^ demonstrated robust *in vitro* activity of lefamulin against multidrug-resistant *M. genitalium*, with MIC values ranging from 0.002 to 0.063 mg/L. However, further *in vivo* studies are warranted to evaluate the efficacy of lefamulin in the treatment of *M. genitalium* infections clinically.

We successfully cultured 50.0% of the collected *M. genitalium* isolates. While other studies have linked successful culture with higher initial bacterial loads, we did not observe significantly lower loads in the specimens where culture attempts were unsuccessful. The main strength of this study is the extensive range of azithromycin concentrations tested on isolates with an A2058T mutation. Previous studies have primarily focused on assessing azithromycin resistance in isolates harbouring the A2058G or A2059G mutations, with limited data available regarding the A2058T mutation.

A limitation of this study is the lack of clinical patient data regarding the treatment efficacy of azithromycin for the collected urine specimens. Without this information, the clinical relevance of the increased *in vitro* susceptibility to azithromycin observed in the A2058T isolates remains uncertain. Additionally, urine samples were collected randomly from men visiting the STI clinic, irrespective of symptom severity. Consequently, not all cultured isolates may have been clinically relevant, as Dutch treatment guidelines recommend treating only patients with persistent urethritis symptoms for at least 4 weeks.^32^ Finally, given the extensive workload involved in these experiments and the challenges associated with culturing *M. genitalium*, we assessed only three concentrations of antibiotics other than azithromycin, rather than using a 2-fold dilution series. Additionally, the sample size of successful cultures for each MRAM SNP was limited.

In conclusion, this study demonstrated that *M. genitalium* isolates exhibit variable *in vitro* growth inhibition induced by azithromycin, depending on the presence of specific MRAM SNPs. This variability may account for differences in clinical azithromycin treatment outcomes observed in patients infected with *M. genitalium* harbouring specific MRAMs. Phenotypic susceptibility testing of alternative antibiotics *in vitro* revealed that second-line treatment options with fourth-generation fluoroquinolones, tetracyclines and lefamulin may be effective against *M. genitalium* infection, including those harbouring an MRAM. With the ongoing increase of macrolide and fluoroquinolone resistance in *M. genitalium* globally, treatment options are becoming increasingly limited, posing a significant public and individual health challenge. This study emphasizes the importance of culturing genetically diverse isolates to associate genotypic with phenotypic antibiotic resistance and to explore alternative oral therapies. Future studies incorporating data on clinical treatment efficacy and symptom severity are essential to identify optimal first- and second-line treatments for *M. genitalium* infections.

## Supplementary Material

dkae430_Supplementary_Data

## Supplementary data

Figure S1 and Table S1 are available as Supplementary data at *JAC* Online.
