# Supplementary material for: Phenotypic antibiotic resistance of Mycoplasma genitalium and its variation between different macrolide resistance-associated mutations
Source: J Antimicrob Chemother. 2024 Dec 4;80(2):465–71. doi: 10.1093/jac/dkae430 (PMC11787896; doi:10.1093/jac/dkae430)
Supplement: dkae430_Supplementary_Data [file dkae430_supplementary_data.docx]

**Supplementary data**


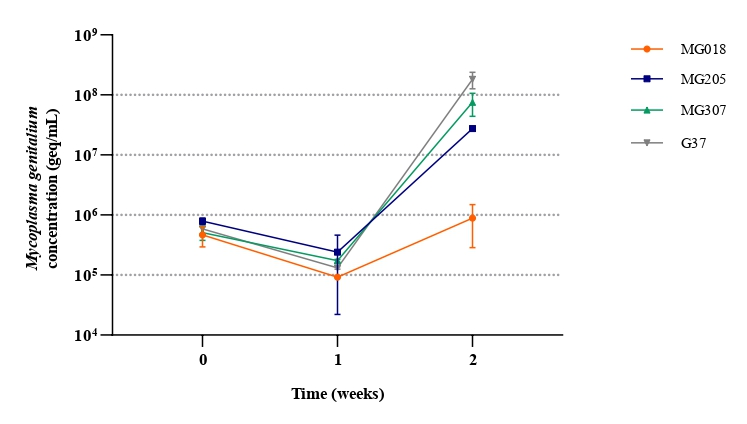


**Figure S1**. Concentration of *M. genitalium* measured in genome equivalents (geq) per mL over a two-week growth period in 12-well plates. Each isolate was tested in triplicate, with supernatant samples taken from each well. Isolates MG018, MG205, and MG307 harbored the MRAMs A2058G, A2058T, and A2059G, respectively.

**Table S1.** MRAM SNPs and MIC values for selected antibiotics across individual *M. genitalium* strains.

| **Strains** | **MRAM** | **AZM**  **(mg/L)** | **LVX (mg/L)** | **MXF (mg/L)** | **STFX (mg/L)** | **MIN (mg/L)** | **DOX**  **(mg/L)** | **SPT (mg/L)** | **LEFA**  **(mg/L)** |
| --- | --- | --- | --- | --- | --- | --- | --- | --- | --- |
| G37 | WT | 0.008^a^ | >4 | 1 | 0.25 | >1 | >2 | >25 | ≤0.004 |
| MG139 | WT | ≤0.016 | >4 | 0.5^a^ | 0.25 | >1 | ≤0.125 | >25 | ≤0.004 |
| MG308 | WT | ≤0.016 | 1 | ≤0.25 | 0.25 | ≤0.25 | ≤0.125 | ≤2.5 | ≤0.004 |
| MG305 | WT | ≤0.016 | ND | ND | ND | ND | ND | ND | ND |
| MG018 | A2058G | >32 | 4 | ≤0.25 | 0.25 | TF | 0.5 | ≤2.5 | 0.004 |
| MG202 | A2058G | >32 | 4 | 1 | ≤0.032 | ≤0.25 | 2 | 12.5 | 0.016 |
| MG326 | A2058G | >32 | ND | ND | ND | ND | ND | ND | ND |
| MG106 | A2059G | 32 | 4 | 1 | ≤0.032 | ≤0.25 | ≤0.125 | 12.5 | ≤0.004 |
| MG168 | A2059G | >32 | 1 | TF | ≤0.032 | 1 | TF | >25 | TF |
| MG209 | A2059G | >32 | 1 | ≤0.25 | ≤0.032 | 0.5 | 0.5 | 25 | ≤0.004 |
| MG210 | A2059G | >32 | TF | TF | TF | TF | TF | TF | TF |
| MG307 | A2059G | >32 | 1 | ≤0.25 | ≤0.032 | ≤0.25 | 0.5 | 12.5 | 0.016 |
| MG037 | A2058T | 8 | >4 | 1 | ≤0.032 | 1 | ≤0.125 | 25 | 0.063 |
| MG182 | A2058T | 8 | >4 | 1 | 0.25 | 1 | 2 | >25 | 0.016 |
| MG205 | A2058T | 8 | TF | TF | TF | TF | TF | TF | TF |
| MG212 | A2058T | 32 | 4 | 1 | ≤0.032 | 0.5 | 2 | 12.5 | ≤0.004 |

^a^Adjusted antibiotic concentrations based on prior characterization of strain. SNPs, single nucleotide polymorphisms; MRAM, macrolide resistance-associated mutations; AZM, azithromycin; LVX, levofloxacin; MXF, moxifloxacin; STFX, sitafloxacin; MIN, minocycline; DOX, doxycycline; SPT, spectinomycin; LEFA, lefamulin; WT, wildtype; ND, note done; TF, test failed.
